# Assessment of thyroid function tests in patients with chronic obstructive pulmonary disease

**DOI:** 10.25122/jml-2022-0069

**Published:** 2022-12

**Authors:** Ali Hasan Ismaeel Alshamari, Falah Deli, Hesham Ibrahim Kadhum, Iman Jabar Kadhim

**Affiliations:** 1 Department of Medicine, College of Medicine, University of Kufa, Najaf, Iraq; 2 Babylon Health Directorate, Alsadiq Teaching Hospital, Babylon, Iraq; 3 Department of Community Medicine, College of Medicine, University of Kufa, Najaf, Iraq

**Keywords:** thyroid function tests, chronic obstructive pulmonary disease, COPD – Chronic obstructive pulmonary disease, FT4 – Free thyroxin, FT3 – Free triiodothyronine, FEV1 – Forced expiratory volume/1second, FSH – Follicular stimulating hormone, GH – Growth hormone, GOLD – Global initiative for chronic obstructive lung disease, IL6 – Interleukin-6, IL1 – Interleukin-1, LH – Luteinizing hormone, NTI – Non-thyroid illness, PFT – Pulmonary function test, TFT – Thyroid function test, TNF-a – Tumor necrosis factor-alpha, TSH – Thyroid-stimulating hormone, TT3 – Total triiodothyronine, TT4 – Total thyroxin, VC – Vital capacity

## Abstract

Chronic obstructive pulmonary disease (COPD), a group of diseases of distinct aetio-pathological consideration with different phenotypic presentations where smoking is the leading cause, all share the ultimate result of airflow limitation. This study aimed to evaluate thyroid function tests (TFT) in patients with COPD. Pulmonary function tests (PFT) were performed for 30 patients with obstructive lung disease and fifteen healthy control individuals. We measured SPO_2_ to confirm COPD and assess the severity of the disease and assessed TT3, TT4, and TSH using the ELISA test. The values of VC, FVC, and FEV in the first second and PEF, TSH, and SPO_2_ were lower in the COPD group than in the control group (P-value=0.001). In severe COPD (FEV_1_<50%), there was a significant reduction in T3 but not T4 or TSH compared to mild-moderate COPD patients. Thyroid dysfunction was observed in patients with COPD pointing to a metabolic response; patients with lower weight indices had a lower TSH and, consequently, T3.

## INTRODUCTION

Despite the numerous measures to decrease smoking prevalence in many communities, especially in developed countries, chronic obstructive pulmonary disease (COPD) remains a significant contributor to chronic morbidity and noticeable mortality worldwide [1]. Most of this problem's phenotypes are characterized by chronic airflow limitation with insidious progression and poor reversibility [2], and the more severe the limitation, the more influenced the survival rate is [3]. Limitation of exercise capacity and, later on, dyspnea are the most important symptoms observed [4]. Diagnosis of COPD generally depends on clinical presentation; however, objective demonstration of airflow obstruction by spirometry is performed where the forced expiratory volume/1second (FEV1) measured after administration of bronchodilator is less than 80% of the predicted value supported by a low FEV1/FVC (<70%) [5]. The severity of COPD is assessed using different guidelines; however, the GOLD classification (the Global Initiative for Chronic Obstructive Lung Disease) is the most reliable and applicable [6]. The GOLD criteria for COPD severity are shown in Table 1.

**Table 1:** Global Initiative for Chronic Obstructive Pulmonary Disease (COPD) [6].

GOLD stage	Severity	Symptoms	Spirometry
**0**	At risk	Chronic cough, sputum production	Normal
**I**	Mild	With or without chronic cough or sputum production	FEV1/FVC<0.7 and FEV1 80% predicted
**II**	Moderate	With or without chronic cough or sputum production	FEV1/FVC<0.7 and 50%<FEV1<80% predicted
**III**	Severe	With or without chronic cough or sputum production	FEV1/FVC<0.7 and 30%<FEV1<50% predicted
**IV**	Very severe	With or without chronic cough or sputum production	FEV1/FVC<0.7 and FEV1<30% predicted or FEV1<50% predicted with respiratory failure or signs of right heart failure

Medical management of COPD is focused fundamentally on the primary problem. However, despite optimal management, the relationship between the primary pulmonary function impairment on the one hand and the general disability on the other is still largely poor. The association with thyroid function impairment was postulated in hypoxemic and hypercapnia COPD patients [7, 8]. Chronic hypoxemia affects various endocrine organs, and derangement of hypothalamic-pituitary function can be evident, but without important changes in the hypothalamic-thyroid axis [9]. Regulation of thyroid hormone is abnormal in many no thyroidal illnesses (NTI); this includes a decrease in total triiodothyronine (TT3) and free triiodothyronine (FT3), normal or decreased total and free thyroxin (TT4 and FT4), and commonly normal levels of thyroid-stimulating hormone (TSH). Patients with critical illnesses are the most affected by these changes, as observed in sepsis, surgery, acute coronary syndromes, and severe starvation [10, 11]. Moreover, these changes occur in other chronic illnesses with diverse presentations, such as heart failure, chronic kidney diseases, connective tissue diseases, and malignancies [11–14].

Furthermore, hormone level abnormalities are largely correlated with the severity of the underlying acute or chronic condition. The activation of the inflammatory cytokine system is associated with the non-variable expression of alterations of thyroid hormone in NTI despite the great diversities of these disorders [15, 16]. The release of IL-6 produced by monocytes/macrophages under the control of many factors is shown to modulate the metabolism of thyroid hormone [17, 18]. Furthermore, the increasing levels of IL-6 have a strong inverse relationship with TT3 and the TT3/TT4 ratio in children with NTI [18, 19]. Thyroid function derangement influences the balance of energy and body composition and the increased energy expenditure at rest [20]. Physical activities that lead to the observed hypermetabolism can affect the nutritional status of patients with COPD [21], and insufficient dietary intake will result in a negative energy balance and may be the major reason for weight loss [22]. TSH serum levels, whether basal or after stimulation, was found to be low in patients with low FEV1 [23].

## MATERIAL AND METHODS

This study was conducted in Alsader-Medical City, Alnajaf, Iraq, between March 2010–March 2011. All patients had a physical examination, and their medical history was assessed. We included 45 individuals further divided into the control group (15 healthy individuals) and 30 patients with clinically stable COPD (irrespective of symptoms of chronic cough, expectoration, and dyspnea). We excluded patients with known respiratory diseases other than COPD (asthma, bronchiectasis), previous known thyroid problems or surgeries, and other endocrine problems. No patients had a history of iodine-containing drugs or other drugs that may disturb thyroid gland function. All patients had post-bronchodilator spirometry in the morning to measure FEV1, peak expiratory flow (PEF), and vital capacity (VC) (Spirolab-II-MIR S/N 504244) to confirm the diagnosis of COPD. Patients were divided into two groups based on FEV1: group 1 – severe COPD (FEV1<50% of predicted values), and group 2 – mild-to-moderate (FEV1>50% of predicted values). SPO_2_ was measured with pulse oximetry to determine blood O_2_ saturation, and T3, T4, and TSH were assessed on the same day just after spirometry using an ELISA test in Al-Najaf Center for Diabetic Research from Al-Sadder Medical City following normal values (T4=4.2–11.0 ug/100ml, T3=0.52–1.6 ng/ml, TSH=0.25–5.0 mu/ml.)

### Statistical analysis

Statistical analyses were performed by SPSS software version 17.0 (SPSS, Chicago). Continuous data were subjected to a normality test (Shapiro Wilk test), and data with normally distribution were presented as mean and standard deviation and analyzed. A P-value<0.05 was considered significant. Values are expressed as mean±SD.

## RESULTS

There was a significant difference in O_2_ saturation, FEV1, and VC between COPD and control groups. Furthermore, there was a significant decrease in TSH levels in the COPD group compared to the control group (p-value=0.001). However, there was no difference in T3, T4, and BMI levels between these two groups (Table 2). According to Table 3, T3 was significantly lower in patients with severe COPD (FEV1%<50%, P-value=0.01). Regarding O_2_ saturation, four patients had O_2_ saturation <92%, and the remainder had O_2_ saturation ≥92%. There were no differences in TFT indicators between these two groups (Table 4). Moreover, there were no differences regarding TFT in the study group, irrespective of other asymptomatic co-morbidities like hypertension, ischemic heart disease, and diabetes mellitus (Table 5). 16 patients had normal weight (BMI=18.5–24.9), 11 were overweight (BMI=25–29.9), and 3 were obese (BMI>30). T3 was higher in obese patients (p-value=0.05) (Table 6). There was no significant correlation between the T3/T4 ratio and COPD severity (Table 7) or degree of hypoxia (Table 8).

**Table 2:** PFT, TFT, O_2_ saturation, and BMI in patients with COPD and control group.

	Study group	NO.	M±STDEV.	P-value
**Age**	COPD	30	61.4±7.0	0.0001
	Control	15	55.2±3.9	
**O_2_ saturation**	COPD	30	93.0±1.6	0.0003
	Control	15	95.3±1.9	
**FEV1**	COPD	30	36.7±16.4	0.0005
	Control	15	90.8±11.2	
**PEF**	COPD	30	28.2±16.5	0.0004
	Control	15	83.3±19.6	
**VC**	COPD	30	48.9±22.1	0.0002
	Control	15	87.3±11.8	
**TSH**	COPD	30	1.3±1.05	0.001
	Control	15	3.4±0.79	
**T3**	COPD	30	0.8±0.33	0.566
	Control	15	0.9±0.18	
**T4**	COPD	30	5.5±1.69	0.240
	Control	15	6.0±0.97	
**BMI**	COPD	30	24.7±4.2	0.742
	Control	15	25.1±3.5	

**Table 3:** TFT in patients with COPD according to COPD severity.

	Severity (FEV1<50%, FEV1≥50%)	NO.	M±STDEV.	P-value
**TSH**	<50%	24	1.2±0.9	0.566
	≥50%	6	1.5±1.4	
**T3**	<50%	24	0.7±0.2	0.01
	≥50%	6	1.1±0.3	
**T4**	<50%	24	5.4±1.7	0.65
	≥50%	6	5.8±1.3	

**Table 4:** TFT in patients with COPD according to O_2_ saturation.

	O_2_ saturation	NO.	M±STDEV	P-value
**TSH**	<92%	4	0.6±0.6	0.198
	≥92%	26	1.4±1.0	
**T3**	<92%	4	0.7±0.2	0.419
	≥92%	26	0.8±0.3	
**T4**	<92%	4	4.9±2.1	0.453
	≥92%	26	5.6±1.6	

**Table 5:** TFT in patients with COPD according to the presence of asymptomatic co-morbidities.

	Comorbidities	NO.	M±STDEV.	P-value
**TSH**	Yes	12	1.3±1.0	0.945
	No	18	1.3±1.0	
**T3**	Yes	12	0.8±0.4	0.472
	No	18	0.9±0.2	
**T4**	Yes	12	5.3±1.8	0.555
	No	18	5.6±1.6	

**Table 6:** TFT in patients with COPD according to BMI.

	BMI	NO.	M±STDEV.	P-value
**TSH**	Normal	16	1.1±0.8	0.526
	Overweight	11	1.4±1.2	
	Obese	3	1.7±1.3	
	Total	30	1.3±1.0	
**T3**	Normal	16	0.7±0.3	0.051
	Overweight	11	0.8±0.2	
	Obese	3	1.2±0.4	
	Total	30	0.8±0.3	
**T4**	Normal	16	5.7±1.8	0.551
	Overweight	11	5.0±1.6	
	Obese	3	6.0±O.6	
	Total	30	5.5±1.6	

**Table 7:** T3/T4 ratio in patients with COPD according to the severity.

	Severity	NO.	M±STDEV.	P-value
**T3/T4**	FEV1<50%	24	0.15±0.071	0.180
	FEV1≥50%	6	0.20±0.075	

**Table 8:** T3/T4 ratio in patients with COPD according to oxygen saturation.

	O_2_ saturation	NO.	M±STDEV.	P-value
**T3/T4**	SPO_2_<92%	4	0.18±0.11	0.380
	SPO_2_≥92%	26	0.16±0.06	

## DISCUSSION

Much controversy exists concerning thyroid function abnormalities and chronic obstructive pulmonary diseases. However, the role of thyroid dysfunction in cachexia associated with COPD still needs further assessment [24]. In this study, there was a significant decrease in the level of TSH in the COPD group compared to the control group, but no significant differences in T3 and T4. Pechatnikov [25] found that T3 and T4 levels were low and TSH levels were high in the COPD group, and a secondary (compensatory) mechanism was claimed to be the cause. A positive association was reported between PaCO_2_ and T3, but no association between TSH and T4 on one side and spirometry values and arterial blood gasses on the other [23]. Clear reasoning for these dissimilarities is not straightforward, and population differences may play a role. In this study, we also found no significant correlation between the T3/T4 ratio and COPD severity or degree of hypoxia. Dimopoulou et al. [22] reported a marked positive correlation between the total T3/total T4 ratio and SPO_2_ and presumed that the severity of hypoxemia was crucial in determining the peripheral metabolism of thyroid hormones. This was not consistent with previous reports [26, 27]. Gow et al. [8] did not find any correlation between arterial blood saturation levels and thyroid hormone measures in COPD patients. In this study, the values of SPO_2_ were correlated with significant changes in T3, and a positive association was found between PFT (FEV1 and VC) and T3, being lower in more severe COPD cases. This finding is consistent with Bratel et al. [7], who found that low FEV1 levels are associated with low TSH levels. Conversely, Pechatnikov et al. [24] found a negative correlation between FEV1 and FT4, with FT4 levels being significantly higher in patients with severe COPD than normal subjects, which is supported by Okutan et al. [26], who found higher T3 levels in patients with severe COPD.

On the other hand, there were no significant associations concerning TSH concentration. Banks and Cooper [25] found no clear relationship between the levels of thyroid hormones and respiratory function in COPD patients, arguing that most of the endocrine dysfunction attributed to COPD was possibly related to factors other than blood gasses changes. The diversity of hormonal changes in COPD patients should point to the fact that COPD must be considered a systemic rather than a sole respiratory disease. Consequently, the manifestations of systems other than the respiratory one must be considered in evaluating their severity, and the treatment follow-up of these manifestations can, to a large extent, change the prognosis of these patients. Interestingly TSH and T3 levels were getting higher with increasing weight in our sample, and this is most likely the effect of TSH suppression with losing weight because of the hypermetabolic state in some patients with low weight and COPD [22, 23].

## CONCLUSIONS

Thyroid dysfunction was observed in patients with COPD pointing to a metabolic response. Patients with lower weight indices had lower TSH and, consequently, T3.
